# A Mini Review of Dolphin Carbohydrate Metabolism and Suggestions for Future Research Using Exhaled Air

**DOI:** 10.3389/fendo.2013.00152

**Published:** 2013-12-16

**Authors:** Sam H. Ridgway

**Affiliations:** ^1^National Marine Mammal Foundation, San Diego, CA, USA; ^2^U.S. Navy Marine Mammal Program, Space and Naval Warfare Systems Center Pacific, San Diego, CA, USA

**Keywords:** dolphin, carbohydrate, breath analysis, diabetes, exhalation, glucose, dog, smell

## Abstract

In the 1960s, I explored some aspects of carbohydrate metabolism in healthy bottlenose dolphins (*Tursiops truncatus*). Their physiological picture resembled what had been described for hyperthyroid diabetics. Dolphins have elevated thyroid hormone turnover, and fasting dolphins maintain a relatively high level of plasma glucose. After dolphins ingest glucose, plasma levels remain high for many hours. Interestingly, plasma glucose must exceed 300 mg/dL (about twice as high as the human threshold) before glucose appears in urine. Due to their diabetes-like states, trainability, and unique natural respiratory anatomy and physiology, dolphins may offer useful clues to metabolites in the breath that may be used to non-invasively monitor diabetes in humans. Dolphins take very rapid and deep breaths that are four or five times as deep as humans and other terrestrial mammals, making them ideal for physiological assessment using non-invasive exhaled air. Avenues for successfully identifying breath-based markers for metabolic disease and physiology in dolphins can be done with both modern technology and the evolutionarily advantageous canine nose. This review summarizes aspects of dolphin metabolism previously learned and offers new directions for diabetes research that may benefit both dolphin and human health.

## Introduction

Bottlenose dolphins (*Tursiops truncatus*), even as neonates, have very low carbohydrate diets. Dolphin milk contains only about 1% carbohydrate. During the nursing period of 1–3 years, the offspring receives large amounts of lipid and protein, but only small amounts of lactose milk sugars. Of the 1% sugar in dolphin milk, 90% is lactose, and only minor amounts of glucose and its metabolites are found (1). Once dolphins are weaned, their diet primarily consists of whole fish of various species that yield a diet high in fat and protein and are almost devoid of carbohydrate (2). The normal dolphin diet is in many ways similar to the ketogenic diet of humans (3). Dolphin dietary protein in the whole fish diet, however, yields amino acids whose metabolic effect is primarily glucogenic rather than ketogenic.

Given a whole fish diet, I was surprised to find relatively high fasting blood sugar levels in dolphins (4). We collected blood samples after 14-h fasting periods. Mean ± SD glucose values for 110 samples from 10 males was 131 ± 36 mg/dL and for 121 samples from 11 females, mean glucose was 127 ± 27 mg/dL. These values for true glucose seemed high, but the findings were consistent in healthy, non-stressed dolphins. One dolphin had several fasting plasma glucose values over 200 mg/dL. Interestingly, concurrent urinalysis showed no rise in urine glucose. The animals had no symptoms of diabetes mellitus such as weight loss, fatigue, or vomiting (a colleague, Dr. David Kenney indicated that he had a diabetic dolphin that he treated with insulin. While the data were never published, Dr. Kenney’s personal observation led us to probe further into dolphin carbohydrate metabolism).

Our studies showed that dolphin glucose tolerance curves were prolonged. After we gave an oral glucose load of 1.75 g/kg body weight, plasma glucose did not return to baseline levels for many hours. Thus, several of our studies showed a prolonged glucose tolerance curve, mimicking a diabetic response. Interestingly, urine glucose values were not elevated after a glucose load. When enough glucose was given to raise plasma glucose above 300 mg/dL, urine glucose increased. The high threshold for glucose excretion is facilitated by a higher plasma osmolality, which in dolphins, is around 335 mOsm/L (5). Humans with diabetes mellitus have increased plasma osmolality (320 mOsm/L) and acid urine, more like dolphins. Thus, the tubular renal threshold for glucose in dolphins is about twice as high as in humans.

During prolonged fasting states, dolphins do not become ketotic. When dolphins were fasted for 72 h, plasma glucose levels remained at pre-fast levels. Even after 72 h, we were not able to sniff acetone or ketone smells in the dolphin breath. Levels of acetone and acetoacetic acid were also not increased in urine. It appeared that dolphins successfully balance protein and lipid metabolism during fasting. As result, glucose is produced by gluconeogenesis in the liver as body protein is metabolized during prolonged fasting. Muscle and other body protein sources were consumed along with body fat (5). Similar lack of ketoacidosis in another marine mammal model, the northern elephant seal, occurs in more extreme circumstances, including 3-month fasting periods (6). Other animal models for diabetes were recently discussed (7).

## Dolphin Thyroid

Thyroid activity changes, including hypo- and hyper-thyroidism have been associated with diabetes in humans (8–10). Hyperthyroid states have been proposed as both the cause of a diabetes-like hyperglycemia and caused by potential injury to pancreatic islet cells. Dolphin thyroid measurements, including plasma levels of thyroxine (T4) and triiodothyronine (T3), have been previously published (5, 11–15). Dolphin T4 levels are slightly higher or similar to human normal values, depending on tests employed, while T3 levels are within the human normal range. Using isotope labeled T3 and T4 injected simultaneously, turnover rates of both thyroid hormones were higher in dolphins than in humans (13). Future research may focus on the potential role and reason of higher thyroid hormone turnover rates in dolphins and associations with hyperglycemia and their natural diabetes-like states. Dolphin T4 levels have been shown to increase after 24 h of fasting (11).

## Dolphin Diving

In our early studies, immunologically measurable insulin in the blood was low, and plasma insulin was slow to increase after an oral glucose load (5). This negligible insulin response to glucose was different than the hyperinsulinemic response after ingestion of high protein fish diets (15). The lack of increased insulin with elevated glucose in dolphins may be especially important since high insulin levels can inhibit gluconeogenesis, and gluconeogenesis is the source of nearly all glucose from the natural fish diet. Lower insulin levels may have some significance as it relates to a dolphin’s diving physiology. Considerable lactate is produced during a dive (16–18). Lactate is well used by the diabetic heart, whereas its glucose utilization is extremely low. When insulin is supplied, the heart increases its utilization of glucose but not of lactate; whereas, brain metabolism of glucose is little affected by insulin (19). Increased lactate consumption by the heart might be advantageous to an animal making frequent dives. The blood glucose level closely follows the lactate level and increases slightly after a dive (20). Trained belugas (*Delphinapterus leucas*) had marked increases in plasma lactate after trained dives in the open ocean (18).

In another study, trained dolphins made numerous dives in the open ocean. Upon returning from depth, animals exhaled in an underwater funnel before taking a breath at the surface (21). After dives to 200 m depth, while the dolphin was at the surface just after the dive, we conducted a short study. The animal was signaled to exhale into the funnel on the third, sixth, and ninth breath after dives. After about nine breaths the dolphin was ready to dive again. I interpreted the results to indicate that it took about nine breaths for the dolphin to blow off sufficient CO_2_ to stay in aerobic metabolism after a 200 m dive. For deeper dives and longer breath holds, exhaled lung air could reach levels of only 1.5% O_2_, suggesting insufficient O_2_ to maintain aerobic metabolism.

From these results, we concluded that an almost complete anaerobicity exists throughout the animal during the last part of a prolonged dive (21). The very low exhaled O_2_ data indicated that the brain must also be operating with at least partial reduction in oxygen consumption during the period. Another diving marine mammal, the harbor seal (*Phoca vitulina*), has an extreme capability for cerebral hypoxia (22). More recently, it was determined that the aerobic dive limit for belugas (*Delphinapterus leucas*) was about 10 min while the animals could dive for 18 min (18). It is known that brain tissue can function anaerobically for a short time if sufficient glucose is available as a substrate for anaerobic metabolism. Therefore, a healthy level of blood glucose is critical for a mammal that can make long dives. This may explain why cetaceans, even as adults, have extremely efficient glucose carrying capacity *via* GLUT-1 transporters; among adults, this capability was previously thought unique to humans and other primates (23).

## Respiratory Quotients and Metabolism of Mono- and Di-Saccharides

**Figure 1:**
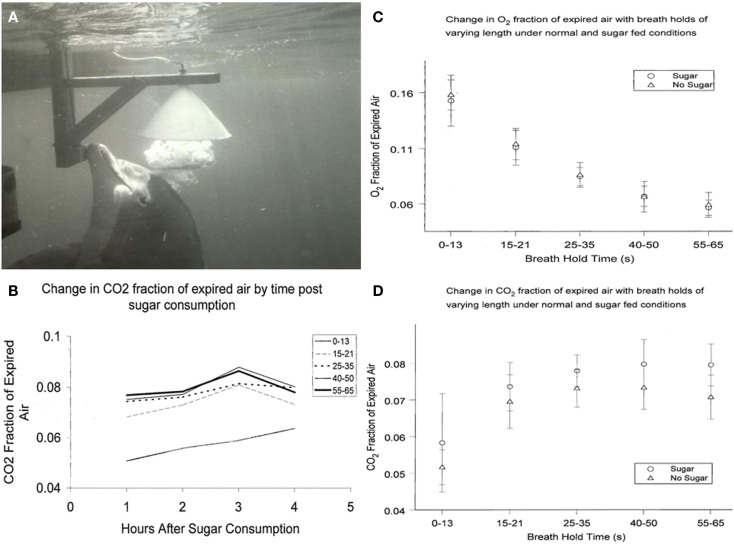
**(A)** Dolphin exhales, exploding a large bubble into the water-filled collecting funnel. **(B)** Change in CO_2_ fraction of exhaled breath from 1 to 4 h after sugar consumption. Breath holds less that about 15 s are not as reliable indicators of CO_2_ total expired. **(C)** Change in O= fraction of expired breath 1–4 h after sugar consumption. **(D)** Change in CO_2_ fraction of expired breath 1–4 h after sugar consumption.

The respiratory quotient (RQ = CO_2_ eliminated/O_2_ consumed) is an indicator of metabolic balance, including the relative oxidation of glucose, protein, and fat. In humans, pure carbohydrate oxidation results in the highest RQ (1.0), while pure fat oxidation results in the lower end RQ (0.7) (24). Human studies have demonstrated that administration of glucose increases the RQ (25). To determine effects of ingested glucose on the RQ in dolphins, we studied CO_2_, and O_2_ exhaled in the breath following a fed glucose load of 1.75 g/kg of body weight. Analysis of the exhaled breath (Figure 1) revealed that the RQ remained close to 0.70, actually lower than when they are fed fish or fasted for short periods. Future research may explore the adaptive relevance of why dolphins do not have an increased RQ in response to glucose administration, including an apparent lack of an oxidative response.

Disaccharidases are enzymes that break ingested disaccharides (i.e., sucrose, lactose, and maltose) into simpler sugars (monosaccharides, including glucose, fructose, and galactose). In humans, insulin deficiency induces an abnormal increase in intestinal disaccharidases (26). Disaccharidase in the gastrointestinal tract is absent in some species of marine mammals (27). Since dolphin intestinal disacchadridase had not been examined, I decided to test the ability of the dolphin to digest sucrose using the exhaled breath.

Results from sucrose ingestion studies were slightly different from earlier studies using glucose. CO_2_ in dolphin breath was measured after oral sucrose ingestion (1 kg of sugar in fish). We analyzed dolphin breath after various periods of breath hold and compared sugar-fed days with days when only fish were fed. Multivariate 2-way analyses of variance were used to evaluate differences in CO_2_ and O_2_ fraction of air expired, as well as respiratory quotients, by ingestion or no ingestion of sugar. Results are shown in Figures 1B–D. Similar to my findings with ingested glucose, there were no significant differences in O_2_ or RQ among dolphins that did or did not eat sugar; or by hours after eating sugar. CO_2_ levels, however, were higher among dolphins that ingested sugar compared to dolphins that did not (0.06 ± 0.013 and 0.05 ± 0.005, respectively; *P* < 0.001). Among human patients, PaCO_2_ conversely decreased when enteral-fed high fat, low carbohydrate diets, demonstrating that dolphins and humans may have similar increases and decreases in CO_2_ in response to higher and lower carbohydrate intake (28). Thus, changing CO_2_ levels with sucrose consumption indicates that dolphins, unlike some marine mammals, can digest complex carbohydrates. This would not be unexpected given that dolphins originated from land and their closest terrestrial, ungulate relatives have diets with complex carbohydrates (29).

Single breath samples from dolphins has been studied to further elucidate diving physiology and carbohydrate metabolism. Recently, nitric oxide (NO) levels in dolphin breath have been determined (30).

## Future Breath Research

Due to the dolphin’s unique respiratory anatomy and their diabetes-like physiology, important results will likely come from dolphin breath and breath condensate measurements (31). Analysis with modern equipment will enable more discriminate detection of compounds linked to changing metabolic states. However, I propose that the intelligence of the canine and its sensitive nose will yield useful data as well.

### The dolphin breath

Dolphins breathe less frequently than terrestrial mammals. They compensate by taking deeper breaths, and they extract more oxygen (O_2_) from the breath (5, 32). In terrestrial mammals, including humans, lung volume represents about 6% of body volume irrespective of body weight (33). In bottlenose dolphins that we have studied, total lung volume on a milliliter per kilogram basis is similar to that of humans. Thus, compared to humans, dolphins take in at least four times as much air with each breath. Additionally, dolphins breathe less frequently, take much deeper breaths, and extract more O_2_ from each lung full of air.

A total of 80% tidal air was measured in dolphins (32). Dolphins inhale in a fraction of a second, hold the breath for a considerable period then rapidly exhale and inhale again. Their blowhole and robust nasal cavities, with no turbinates or other structures for potential slowing of the air, allow for rapid inhalation through a short robust trachea to the depth of each lung. Dolphins take in air that is almost 21% O_2_. At the end of a long breath hold, dolphin exhaled air can contain as little as 1.5% O_2_. The alveoli of the dolphin lung are structured for maximum exposure of lung air to blood circulation. The inter-alveolar septa have double capillary beds separated by an elastic-tissue membrane (34). Compared to a single capillary bed in human lung, the dolphin lung may offer a more efficient exchange of O_2_ and carbon dioxide (CO_2_). In addition, other metabolites can be efficiently exchanged between blood and lung air (35).

### Collecting dolphin breath

Wondering how to collect the dolphin breath, as I walked beside a dolphin pool, an animal approached me and released a large bubble. This was a common dolphin behavior. They sometimes exhale large bubbles under aggregations of fish very near the surface. The rising bubble carries some of the fish to the surface immobilizing the fish just long enough for the dolphin to easily take them. For me, the bubble release just below me appeared to be a gesture connected with food (35). Trainers used this natural bubble blowing behavior, and found that dolphins readily exhaled into a large water-filled, underwater funnel (21, 30). After an explosive exhalation, dolphin breath displaced the water in a fraction of a second and flowed through tubing for collection and analysis (Figure 1A).

### Canine detectors

**Figure 2:**
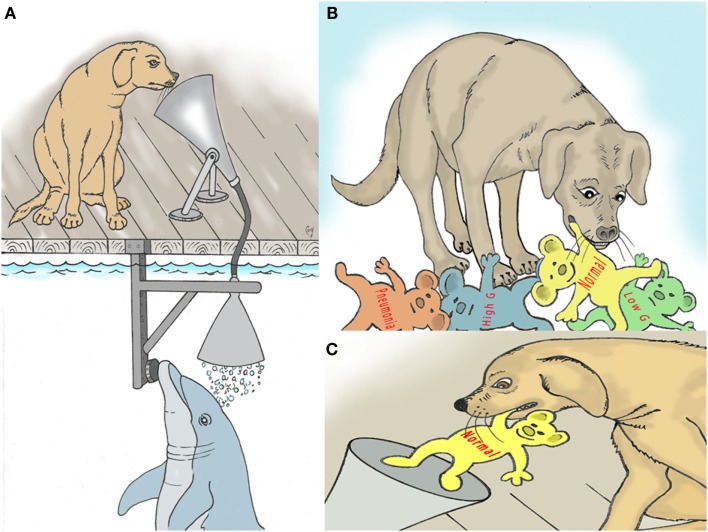
**(A)** Depiction of trained dog sniffing dolphin breath for assessment of metabolic and disease markers. **(B)** Dog selects named models that represent various breath markers of metabolism and disease. **(C)** The selected model is deposited back into the “sniff” funnel to confirm the dog’s decision.

It is well known that canines (dogs) have a very sensitive sense of smell (36). They also have a very good ability to learn the names of numerous different objects (37, 38). This cognitive and sensory ability can be melded using techniques employed to train dolphins. Dolphins will exhale into an underwater funnel, the breath with flow up to the dog for detection of breath markers by smell (Figure 2). The dog then goes to pick up a specific marker that has been identified by word and by odor to match a breath marker of interest, for example acetone (Figures 2B,C). Dogs have also been trained to communicate using a keyboard (39). The keyboard capability might also be used.

### Advantage of canine detectors

Although sophisticated analysis equipment is available in the laboratory setting, it is not readily adaptable for use at the dolphin locations. For example, seawater is often corrosive to complex electronic equipment. Dolphin breath has been collected in special bags and removed to the laboratory for analysis. In some cases, this may work well (30). In many cases, however the immediate feedback from the canine detector would be invaluable. In addition, canines are quite mobile and can be posted at sea or in remote locations where laboratory support and logistics are difficult.

In summary, while the bottlenose dolphin can have a sustained postprandial hyperglycemia, mimicking diabetes in people, dolphins appear to have many adaptations that enable the benefits of readily available glucose while potentially protecting them against complications of hyperglycemia. Understanding how these mechanisms work may have profound benefits for human health, especially with regard to diabetes.

## Conflict of Interest Statement

The author declares that the research was conducted in the absence of any commercial or financial relationships that could be construed as a potential conflict of interest. The Guest Associate Editor, Stephanie Venn-Watson declares that, despite being affiliated with the same institution as the author Sam Ridgway, the review process was handled objectively and no conflict of interest exists.
